# The effect of muscle ultrastructure on the force, displacement and work capacity of skeletal muscle

**DOI:** 10.1098/rsif.2023.0658

**Published:** 2024-05-22

**Authors:** Nihav Dhawale, David Labonte, Natalie C. Holt

**Affiliations:** ^1^ Department of Evolution, Ecology and Organismal Biology, UC Riverside, Riverside, CA, USA; ^2^ Department of Bioengineering, Imperial College London, London, UK

**Keywords:** actin, myosin, invertebrate, musculoskeletal dynamics, muscle work density, LAMSA

## Abstract

Skeletal muscle powers animal movement through interactions between the contractile proteins, actin and myosin. Structural variation contributes greatly to the variation in mechanical performance observed across muscles. In vertebrates, gross structural variation occurs in the form of changes in the muscle cross-sectional area : fibre length ratio. This results in a trade-off between force and displacement capacity, leaving work capacity unaltered. Consequently, the maximum work per unit volume—the work density—is considered constant. Invertebrate muscle also varies in muscle ultrastructure, i.e. actin and myosin filament lengths. Increasing actin and myosin filament lengths increases force capacity, but the effect on muscle fibre displacement, and thus work, capacity is unclear. We use a sliding-filament muscle model to predict the effect of actin and myosin filament lengths on these mechanical parameters for both idealized sarcomeres with fixed actin : myosin length ratios, and for real sarcomeres with known filament lengths. Increasing actin and myosin filament lengths increases stress without reducing strain capacity. A muscle with longer actin and myosin filaments can generate larger force over the same displacement and has a higher work density, so seemingly bypassing an established trade-off. However, real sarcomeres deviate from the idealized length ratio suggesting unidentified constraints or selective pressures.

## 1. Introduction

**Figure 1.**
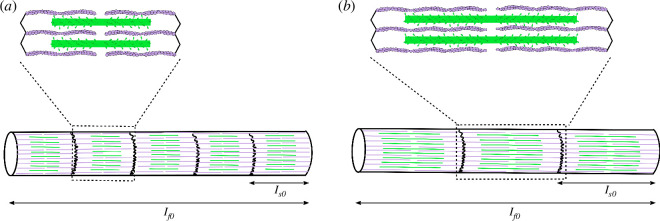
Schematic showing the structure of actin (purple) and myosin (green) filaments and their organization into sarcomeres (top) and myofibrils (bottom). The sarcomere is bounded by Z-discs and the bare zone, absent of heads, in the centre of the myosin filament can be seen. Short (***a***) and long (***b***) contractile protein filament lengths are shown, and the effect of varying optimum sarcomere length ( $l_{s0}$ ) on the number of sarcomeres in myofibril or muscle fibre of a given length ( $l_{f0}$ ) can be seen.

Skeletal muscle generates the mechanical output required for the majority of animal movements through interactions between the contractile protein filaments: actin, myosin and titin. Considerable variation in muscle and organismal performance is exhibited across muscles and species, and this diversity is underpinned by variation in muscle structure and physiology [1–5]. For example, leaf-cutter ants display structural adaptations in their mandibular closer muscle that enable generation of the high forces required for cutting [6], and birds have long pectoralis muscle fibres [1] with fast myosin isoforms [7] to satisfy the substantial work and power requirements of flight. Variation in muscle performance, and the constraints that bind it [8], are key components of fitness [3,9], making skeletal muscle an excellent system in which to study the relationship between subordinate traits, limits to organismal performance, and fitness [10,11]. Muscle performance is an emergent property that arises from at least three hierarchical elements: (i) the molecular agents of the contraction itself; (ii) the ultrastructural arrangement of these agents into the smallest contractile unit, the sarcomere; and (iii) the gross architectural arrangement of sarcomeres into muscle fibres and muscles [12] (figure 1). Thus, muscle provides not only the opportunity to study the relationship between subordinate traits, performance and fitness but also the role of the emergent properties that arise from the interaction of subordinate traits across scales in this relationship.

At the molecular level, cross-bridge interactions between the actin filament, a helical polymer [13], and the myosin filament, a bipolar helical polymer containing myosin dimers whose tails are anchored in the centre of the filament and heads project outwards [14], consume chemical energy to generate muscle force, work and power. Titin, a tunable viscoelastic spring, can contribute to these mechanical outputs when stretched [15–19]. At the ultrastructural level, actin, myosin and titin filaments are organized into sarcomeres in which actin filaments project from the Z-discs and myosin filaments are anchored in the centre of the sarcomere by titin [20]. When viewed in three-dimensions, these overlapping contractile protein filaments created a highly ordered lattice [21,22]. At the gross architectural level, sarcomeres are organized in series into myofibrils, myofibrils are arranged in parallel in muscle fibres, and muscle fibres are organized into muscles [12]. This hierarchical arrangement of muscle, and the molecular interactions that underpin muscle contraction, have downstream effects on the mechanical performance space accessible to muscle [12,23].

Upon muscle activation, myosin heads cyclically bind to actin forming cross-bridges [24], and titin likely binds to actin [25–27]. Cross-bridges undergo force-generating conformational changes that slide actin past myosin, as described by the cross-bridge and sliding-filament theories [24,28,29], and potentially wind titin onto actin, as described by the winding-filament hypothesis [25,27]. Cross-bridge forces act to shorten the sarcomere [28], increasing lattice spacing [30,31]. These mechanisms of contraction give rise to, among other features [17,18,32], relationships between sarcomere force and shortening velocity and sarcomere force and length. Generation of force by cross-bridges with intrinsically limited cycling rates result in an inverse relationship between shortening velocity and force [33,34], and each myosin isoform has a maximum shortening velocity [35]. Generation of force using interactions between overlapping actin and myosin filaments means that maximum isometric force is only generated at an optimal sarcomere length corresponding to optimal actin–myosin overlap, and force gradually declines to zero at deviating lengths. This results in a limited range of sarcomere lengths over which force can be generated [36].

The hierarchical nature of muscle means that sarcomeric force, displacement and velocity are directly related to gross muscle force, displacement and velocity and that the sarcomeric force–length and force–velocity relationships give rise to fibre and muscle force–length and force–velocity relationships [37]. Hierarchical design and performance are thus intrinsically linked. A natural question, then, is to what extent variations in muscle architecture lead to variations in muscle performance, and whether there exist hierarchical configurations that are best suited for the maximization of specific mechanical output parameters. Here, we explore this question focussing on the arrangement of actin and myosin filaments along the length of a sarcomere and the arrangement of these sarcomeres into muscle fibres.

The amount of force muscle can produce during isolated isometric and shortening contractions depends on the product of the force per cross-bridge, determined by cross-bridge biochemistry, and the total number of cross-bridges that are mechanically in parallel. The number of cross-bridges within a muscle fibre that are mechanically in parallel is determined by the structure of the myosin filament within sarcomeres, and the organization of sarcomeres within the fibre. Consider one-half of a sarcomere; the myosin dimers are individually anchored to the centre of the thick filament, and this anchoring provides the reaction force that ensures local equilibrium; the forces exerted by individual cross-bridges in this half are consequently independent and thus add (figure 1) [38,39]. In contrast, the force between two sarcomere halves or equivalently between two neighbouring sarcomeres needs to be equilibrated at their connection, i.e. at the M-line in the centre of the sarcomere or the Z-discs between sarcomeres. The net fibre force capacity is thus determined by the number of cross-bridges per half sarcomere, which is, in turn, dictated by actin and myosin filament length and overlap, and the number of sarcomeres in the muscle’s cross-section [5,38,40]. The maximum shortening velocity of muscle, in turn, depends on the speed of each cross-bridge, determined by myosin isoform, and the number of sarcomeres that are mechanically in series, i.e. arranged end-to-end in a muscle fibre [38]. The number of sarcomeres in series is determined by sarcomere length, which is determined by actin and myosin filament length and overlap, and fibre length (figure 1).

Myosin isoform, actin and myosin filament lengths, and gross muscle architecture vary across muscles, and these variations have been associated with specialization for different aspects of performance [1–5]. Vertebrate muscle has a highly conserved muscle ultrastructure [41–47]. Hence, performance is determined primarily by myosin isoform and gross muscle architecture. Faster myosin isoforms increase maximum shortening velocity [48], increasing muscle physiological cross-sectional area increases force [5,49], and increasing fibre length increases displacement capacity and maximum shortening velocity [5]. In contrast, invertebrate muscle varies not only in gross structure and myosin isoform but also in ultrastructure [2,45,50]. Actin and myosin filament lengths, and therefore, optimum sarcomere length can differ by more than an order of magnitude. Increasing actin and myosin filament lengths increases the number of cross-bridges mechanically in parallel, and thus force and stress capacity [46,51–54]. However, it also reduces the number of cross-bridges in series [38], and thus shortening velocity [55,56]. Hence, variations in either gross muscle architecture or muscle ultrastructure that increase force capacity do so at the expense of shortening velocity and vice versa. This architectural ‘force–velocity’ trade-off indicates that the instantaneous muscle power per muscle volume, the dot product of force and velocity, is independent of muscle structure and instead depends solely on myosin isoform [4,35].

How muscle ultrastructure affects muscle fibre displacement capacity, the range of lengths over which isometric force can be generated, and work capacity, the maximum dot product of force and displacement during an isolated shortening contraction, is less clear. In vertebrates, where ultrastructure is consistent, force and displacement capacity trade-off in the same way as force and velocity for the same reasons. Myosin isoform typically does not affect either work or displacement capacity, and the maximum work a unit volume of muscle can produce during an isolated shortening contraction is consequently often treated as an invariant constant (e.g. [57–60]). In invertebrates, variation in actin and myosin filament lengths alters the number of sarcomeres in-series, which may alter muscle displacement capacity. However, such ultrastructural variation will also alter the sarcomere displacement over which these actin and myosin filaments overlap, and thus the displacement over which the force drops by a fixed percentage (i.e. the ‘width’ of the force–length curve). This variation in sarcomeric force–length curve width may offset the displacement reduction associated with the reduction in cross-bridges in-series, and so present an opportunity to increase muscle work capacity. Here, we assess how variations in muscle ultrastructure, namely actin and myosin filament length, affect downstream mechanical output at the fibre level.

## 2. Methods

We use a minimalist, one-dimensional model of muscle fibres based on the cross-bridge [24] and sliding-filament theories of contraction [28,29], that ignores titin and the three-dimensional lattice structure of muscle [3,15,30,61], to predict sarcomeric stress–length curves for a range of actin and myosin filament lengths. We then integrate these predictions with the hierarchical structure of muscle to assess how variations in actin and myosin filament lengths affect the force, displacement and work capacity of muscle fibres downstream.

### 2.1. Sarcomeric stress–length relationship

**Figure 2.**
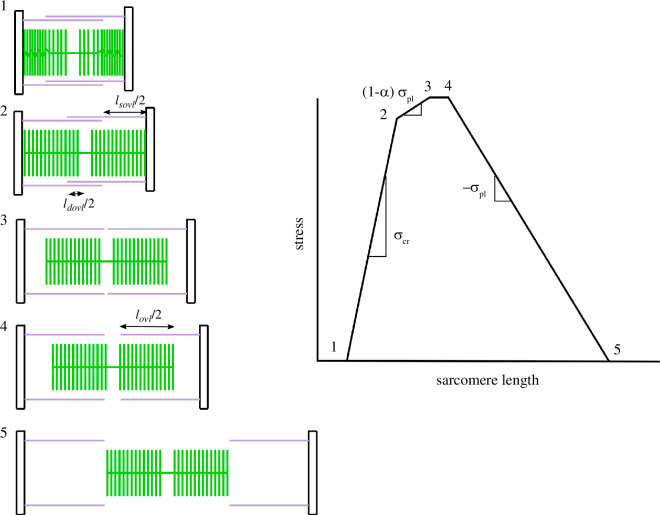
Schematic depiction of how actin (purple) and myosin (green) lengths can be used to predict a sarcomeric stress–length curve with the sarcomere configurations at the inflection points of the curve highlighted. At point 5, there is no overlap between actin and myosin resulting in zero stress. Stress rises between point 5 and point 4 according to the increase in overlap ( $l_{ovl}$ ) and the stress per unit overlap ( $\sigma_{pl}$ ). Maximum stress is reached at point 4 when all myosin heads have access to actin binding sites, and this stress is sustained until point 3 due to the absence of myosin heads in the bare zone (length, $l_{h}$ ). Between point 3 and point 2, force declines according to the empirically determined parameter α , attributed to interference from the opposing actin in the region of double overlap ( $l_{dovl}$ ). Stress then declines to 0 at point 1 according to the empirically determined parameter $\sigma_{cr}$ , attributed to filament crumpling.

In predicting the sarcomeric stress–length relationship, we build on previous work [36,40,43] which models the sarcomeric force capacity based on the overlap between actin and myosin filaments, of lengths $l_{a}$ and $l_{m}$ , respectively, and the interaction of these filaments with each other and the Z-disks. This framework splits the stress–length curves into four sections, delineated by five characteristic points (figure 2). We use four empirically derived parameters that describe the consequences of this filament overlap and interactions (table 1). The model is one-dimensional, and force rather than stress is its natural output. However, for reasons of consistency with the empirically derived parameters [36], and to enable comparison to empirical studies [46], we assume that sarcomere cross-sectional area is independent of actin and myosin filament lengths, and consider stress ( σ , see §4).

**Table 1.** Parameter values for standard variables.

variable	value	source
$l_{h}$ ; length of bare zone of myosin	0.17 μm	[43,62]
α ; drop in stress per unit double-overlap of actin filaments	0.33	[36]
$\sigma_{pl}$ ; stress generated per unit length of overlap	17.7 N cm^−2^μm^−1^	[36]
$\sigma_{cr}$ ; drop in stress per unit length due to crumpling of actin/myosin	54.0 N cm^−2^μm^−1^	[36]

For a fully stretched sarcomere (figure 2, point 5), there is no overlap between actin and myosin filaments, and the stress is consequently zero. At shorter sarcomere lengths (figure 2, section 5–4), the rise in stress is determined by the overlap length between actin and myosin ( $l_{ovl}$ ) and the stress per unit overlap length ( $\sigma_{pl}$ ) (equation (2.1)). Eventually, all myosin heads have access to actin binding sites, and the stress is maximal ( $\sigma_{4}=\sigma_{max}$ ) (figure 2, point 4) (equation (2.2)). Within the bare zone (figure 2, section 4–3), of length $l_{h}$ , the number of myosin heads that have access to actin binding sites, and thus the stress, is constant, resulting in a plateau. We define the optimum sarcomere length ( $l_{s0}$ ) as the centre of this plateau.

At even shorter sarcomere lengths, there are two distinct regions of overlap: a single overlap region, as before ( $l_{sovl}$ ), and a double overlap region ( $l_{dovl}$ ) where the actin filaments from opposing directions cross each other (figure 2, section 3–2). Because actin–myosin bonds are directional, we assume that myosin cannot form force-producing bonds with the opposing actin filament. As in Otten [40], we interpret this ‘crowding’ of actin in the double-overlap region as a reduction of the binding probability of myosin with its ‘home’ actin, which results in a proportional drop in stress per unit double-overlap ( α; table 1) (but see [61]). The extent to which double-overlapped actin filaments interfere with binding probability is parametrized by 0 < α < 1 (equation (2.3)): for α=0 , there is complete interference between actin–myosin bonds in the double-overlap region and thus no stress can be produced; for α=1 , there is no interference and stress is maximal. As the sarcomere continues to shorten, the end of the myosin filament eventually reaches the Z-disc that bounds the sarcomere (figure 2, point 2). It has been suggested that further shortening leads to crumpling of myosin, causing a sharp drop to zero stress (figure 2, section 2–1) (but refer to [63,64]), at a rate that is a function of sarcomere length, as parametrized by $\sigma_{cr}$ (equation (2.4), table 1). If actin length is greater than myosin length $\left(l_{a}>l_{m}\right)$ , then the actin filament ‘crumples’ first. In this case, $l_{2}=l_{a}$ and $\sigma_{2}=\sigma_{3}-\left(1-\alpha \right)\sigma_{pl}\left(l_{a}-l_{h}\right)$ . For the sake of simplicity, we assume that the rate of force drop is identical irrespective of whether actin or myosin crumples. Thus, the stress in the different regions of a sarcomeric stress–length relationship is described by:


(2.1) $$\sigma_{5-4}=\sigma_{pl}\;l_{ovl},$$

(2.2) $$\sigma_{4-3}=\sigma_{4},$$

(2.3) $$\sigma_{3-2}=\alpha \;\sigma_{pl}\;l_{dovl}+\sigma_{pl}\;l_{sovl},$$

(2.4) $$\sigma_{2-1}=\sigma_{2}+\sigma_{cr}\;\left(l_{s}-l_{2}\right),$$

where


(2.5) $$l_{5}=2l_{a}+l_{m},\;\sigma_{5}=0,\;l_{ovl}=l_{5}-l_{s},$$

(2.6) $$l_{4}=2l_{a}+l_{h},\;\sigma_{4}=\left(l_{m}-l_{h}\right)\;\sigma_{pl},$$

(2.7) $$l_{3}=2l_{a}-l_{h},\;\sigma_{3}=\sigma_{4},\;l_{sovl}=l_{s}+l_{m}-2l_{a\;},\;l_{dovl}=l_{3}-l_{s},$$

(2.8) $$l_{2}=l_{m},\;\sigma_{2}=\sigma_{3}+\left(1-\alpha \right)\sigma_{pl}\;\left(l_{m}-l_{3}\right),$$

(2.9) $$l_{1}=l_{2}-\sigma_{2}/\sigma_{cr},\;\sigma_{1}=0.$$

From the sarcomeric stress–length curves, peak stress and curve width can be determined. The width of the sarcomeric stress–length curve was defined as the sarcomere length range spanned by the curve at three different relative stress levels, 0.75, 0.5 and 0.25 $\sigma_{max}$ , and serves as a proxy of the displacement capacity of the sarcomere.

### 2.2. Actin and myosin filament lengths

**Figure 3.**
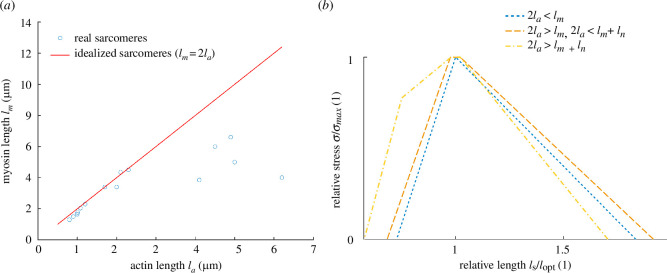
Variation in actin and myosin filament lengths and the resulting 'shape’ of the sarcomeric stress–length curve. (***a***) Actin versus myosin length for real (blue circles) [45,62,65–79] and idealized (red line) sarcomeres. (***b***) Plots of normalized stress ( $\sigma /\sigma_{max}$ ) versus relative sarcomere length ( $l_{s}/l_{s0}$ ) showing how changing $l_{m}/l_{a}$ ratio alters the shape of the stress–length curve.

We use this model to predict sarcomeric force–length curves for experimentally determined actin and myosin filament lengths for real sarcomeres, and for what we refer to as ‘idealized’ sarcomeres with a range of optimum lengths. For the former, we used data from 16 muscles [62,65–79], largely collated by Shimomura *et al*. [45], for which there are published values of $l_{m}$ and $l_{a}$ (figure 3*a*). These muscles include the frog sartorius, cockroach femur and lobster claw closer muscles that we highlight throughout as illustrative examples of muscles with short, medium and long sarcomeres. We define idealized sarcomeres as having a constant $l_{m}/l_{a}$ ratio, $l_{m}={2l}_{a}$ (figure 3*a*). In such a sarcomere, myosin contacts the Z-disc as opposing actin filaments make contact in the centre of the sarcomere. These idealized sarcomeres maximize peak stress for a given total contractile protein filament length, provided that the length of the bare zone, $l_{h}$ , is small relative to actin and myosin filament lengths (table 1). To understand why $l_{m}={2l}_{a}$ is an idealized design that maximizes peak stress, consider first sarcomeres where $l_{m}>2l_{a}$ . Peak stress for such a sarcomere is achieved when all actin binding sites are available to the myosin heads, but not all myosin heads would have an accessible actin-binding site. Peak stress is then determined by actin instead of myosin length as assumed by equation (2.6) ( $\sigma_{max}=2l_{a}\sigma_{pl}<l_{m}\sigma_{pl}$ ). If instead $l_{m}<2l_{a}$ , peak stress drops because there are overall fewer myosin heads in the sarcomere.

The relative lengths of actin and myosin have implications for the shape of the stress–length curve. The general model generates four distinct sections (figure 2, yellow curve in figure 3*b*). However, variations in the $l_{m}/l_{a}$ ratio can result in a change in the ‘shape’, resulting in stress–length curves with three or even two segments (figure 3*b*). If actin filaments reach the bare-zone but are too short for a double-overlap zone, i.e. $2l_{a}>l_{m}$ and $2l_{a}<l_{m}+l_{h}$ , myosin filaments crumple after shortening beyond the plateau, and the stress–length curve has only three segments (orange curve in figure 3*b*). If the combined actin filament length is shorter than the contractile region of the myosin filament, i.e. $2_{la}<l_{m}-l_{h}$ , then there is no plateau and peak stress is determined by actin length, i.e. $\sigma_{max}={2l}_{a}\sigma_{pl}$ ; the stress–length curve then has only two segments (blue curve in figure 3*b*). From the limited data available [45,62,65–79], it appears as though real sarcomeres conform to the idealized sarcomere design at short optimum sarcomere lengths, but deviate from it at longer optimum sarcomere lengths such that $l_{m}<2l_{a}$ (figure 3*a*, and see §4).

### 2.3. Fibre stress–length curves

A key aim of this study is to understand the effect of muscle ultrastructure on gross muscle performance. Thus, we next assess how sarcomeric stress–length curves scale up to fibre stress–lengths curves. To this end, we model a muscle fibre as a sequence of sarcomeres in-series. Consider two fibres of equal length but comprised of sarcomeres of different optimal lengths (figure 1). Since all sarcomeres are in series, fibre stress and sarcomere stress are equal, but fibre displacement is the sum of all sarcomere displacements. Thus:


(2.10) $$\frac{\Delta l_{f}}{l_{f0}}=\frac{\Delta l_{s}}{l_{s0}}$$

(2.11) $$⟹l_{s}=l_{s0}\;\left(1+\frac{\Delta l_{f}}{l_{f0}}\right).$$

Here, $l_{f0}$ and $l_{s0}$ refer to the optimal lengths of the fibre and sarcomere, respectively. Equation (2.11) defines a unique relationship between sarcomere length $l_{s}$ and fibre strain $\frac{\Delta l_{f}}{l_{f0}}$ ; fibre stress–length curves can now be constructed from sarcomere stress–length curves.

Where muscle is used to drive movement, muscle work may be a more pertinent metric of performance than muscle stress or displacement capacity. Stress and curve width can be combined to give work capacity as a natural third metric for muscle performance. The work capacity per unit volume $\left(W_{v}\right)$ , the muscle work density, of a muscle fibre reads as:


(2.12) $$W_{v}=\frac{1}{l_{f0}}\int \sigma_{f}\;\left(l_{f}\right)dl_{f},$$

where the upper and lower limits of the displacement integral determine the strain range over which work density is defined. We report work density for a range of strain levels, with strain centred around the optimal sarcomere length [80,81]. Implicit in this definition is the assumption that the contraction is quasi-static, such that the work output only depends on the stress–length, but not on the stress–velocity, properties.

## 3. Results

We describe sarcomeric and fibre stress–length curves for muscles with varying ultrastructure for both real and idealized ( $l_{m}={2l}_{a}$ ) sarcomeres. From these curves, we extract sarcomeric stress and displacement capacity. We then scale these sarcomeric curves up to muscle fibre stress–length curves and consider the resulting fibre stress, strain capacity and work density.

### 3.1. Sarcomeric stress–length curves

**Figure 4. F4:**
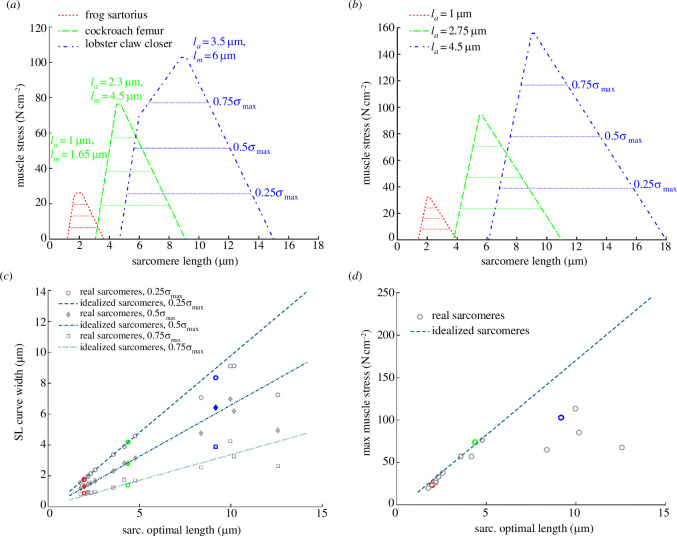
Sample sarcomeric stress–length curves for short (red), medium (green) and long (blue) sarcomeres with (***a***) real filament lengths [45,62,65–79] for the frog sartorius, cockroach femur and lobster claw closer and (***b***), idealized sarcomeres of approximately equal actin and myosin filament lengths. The width of the stress–length curve at 0.75, 0.5 and 0.25 of peak stress are indicated. Longer sarcomeres have longer optimal lengths, (***c***) broader sarcomeric stress–length curves and (***d***) higher peak stresses. The frog sartorius, cockroach femur and lobster claw closer muscles from (***a***) are highlighted in summary figures (*c,d*).

Sarcomeric stress–length curves reveal that increasing actin and myosin filament lengths increase peak stress and the width of the sarcomeric stress–length curve for both real and idealized sarcomeres (figure 4). Peak predicted stress values for the frog sartorius, cockroach femur and lobster claw closer sarcomeres are 25.8, 78.8 and 105.2 N cm^−2^, respectively. However, the deviation in $l_{m}/l_{a}$ ratio from $l_{m}={2l}_{a}$ in real sarcomeres (figure 3*a*) results in a less rapid increase in stress with optimum sarcomere length than predicted for idealized sarcomeres (figure 4*d*). For example, an idealized sarcomere with the same actin length $l_{a}$ as the lobster claw closer muscle would generate a stress of 155.8 N cm^−2^, almost 50% higher than the value predicted from the measured filament lengths. In some cases, this deviation from $l_{m}={2l}_{a}$ is so great that increases in optimum sarcomere length do not result in any increase in muscle stress. For example, there is a nearly threefold increase in optimum sarcomere length in the crayfish extensor carpopoditi muscle [72] compared to the cockroach femur muscle [70,71], yet stress shows a slight decrease (70.8 N cm^−2^ compared to 80.0 N cm^−2^). Hence, although increasing actin and myosin filament, and optimum sarcomere, lengths often increases predicted stress, this is not always the case. Predicted stress depends also on the $l_{m}/l_{a}$ ratio.

The predicted width of the sarcomeric stress–length curve at 0.5 $\sigma_{max}$ is 1.5, 3.1 and 6.4 µm for the frog sartorius, cockroach femur and lobster claw closer sarcomeres, respectively. Width appears to increase slightly more rapidly for idealized than for real sarcomeres, but this effect is much less pronounced than for stress. Stress–length curves for idealized sarcomeres are simply scaled versions of one another (figure 4*b*), but those for real sarcomeres exhibit changes in shape (figure 3) due to differences in the $l_{m}/l_{a}$ ratio (figures 3 and 4*a*). However, despite this variation in shape, the prediction that curve width increases with actin and myosin filament length appears insensitive to the stress level at which the width is calculated (figure 4*c*).

### 3.2. Fibre stress–length curves

**Figure 5. F5:**
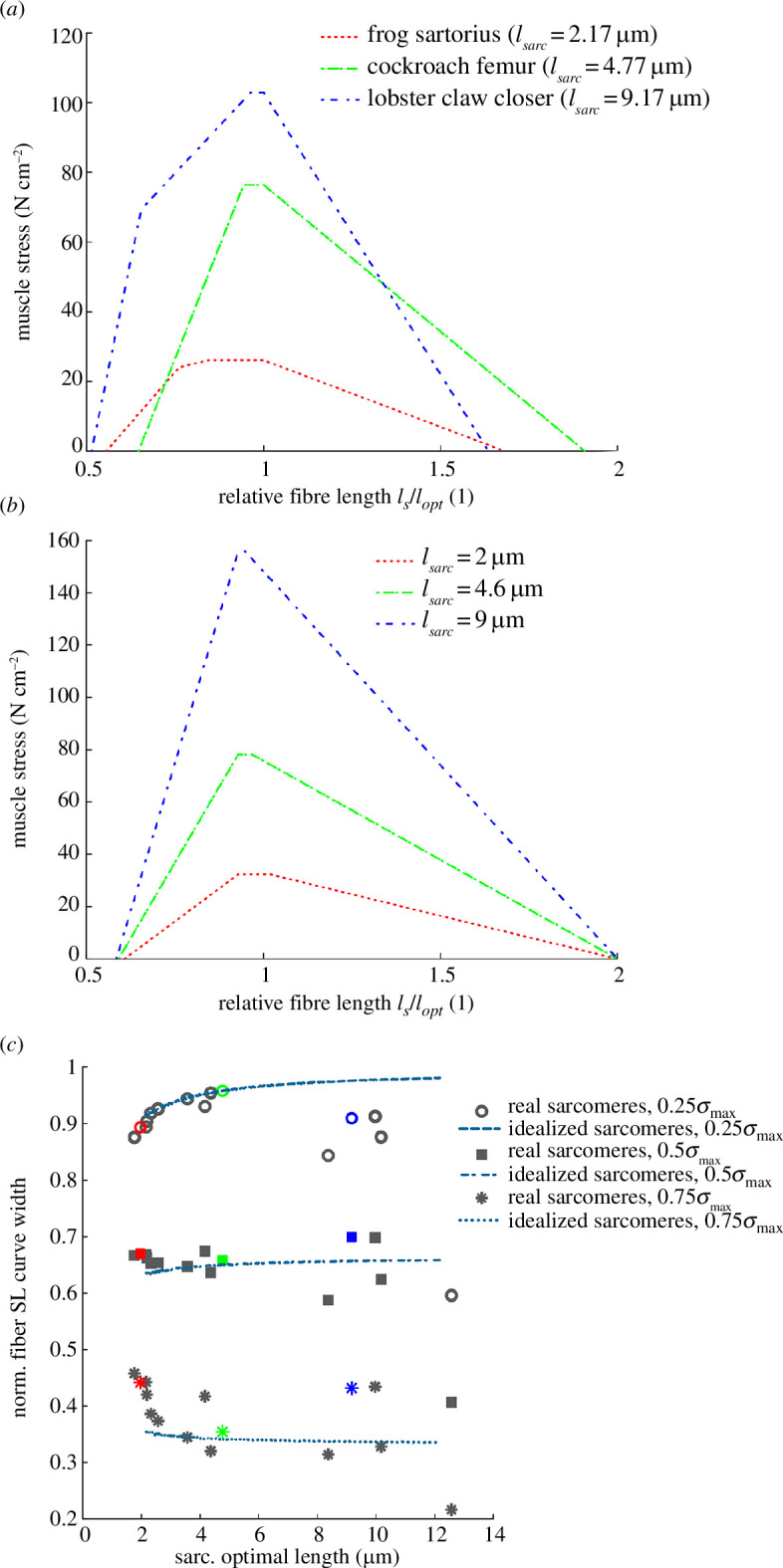
Sample fibre stress–length curves for fibres comprised of short (red), medium (green) and long (blue) sarcomeres with (***a***), real filament lengths [45,62,65–79] for the frog sartorius, cockroach femur and lobster claw closer muscles and (***b***), idealized sarcomeres of approximately equal lengths. The width of the fibre stress–length curves at 0.75, 0.5 and 0.25 of peak stress are indicated. The width of the fibre stress–length curve is relatively insensitive to sarcomere length for both idealized (dashed lines) and real (symbols) sarcomeres (***c***). The frog sartorius, cockroach femur and lobster claw closer muscles from (***a***) are highlighted in summary figure (***c***).

We model a muscle fibre as a number of sarcomeres in series (figure 1). As a direct consequence, any variations in stress observed in sarcomeric stress–length curves are mirrored in fibre stress–length curves, and muscle fibres with longer optimum sarcomere lengths generate higher peak stresses (figure 5*a,b*). However, in contrast to the sarcomeric stress–length curve, there is little to no variation in the fibre stress–length curve width with increasing optimum sarcomere length for either real or idealized sarcomeres (figure 5). Fibres with longer sarcomeres have wider sarcomeric stress–length curves (figure 4). However, each sarcomere also undergoes a larger absolute length change for a given fibre strain compared to fibres with shorter sarcomeres. For sufficiently long actin and myosin filament lengths, where the length of the bare zone is negligible, all sarcomeres with a fixed $l_{m}/l_{a}$ ratio have the same fibre stress–length curve width. For very short sarcomeres, the length of the bare zone is comparable to actin and myosin filament lengths, causing a drop in stress–length curve width and deviation from the linear trend at short actin and myosin filament lengths (lines figure 5*c*). The variability in fibre stress–length curve width for real sarcomeres (blue circles in figure 5*c*) is a consequence of changes to the $l_{m}/l_{a}$ ratio, and so the shape of the stress–length curve, with increasing actin and myosin filament length (figure 3*a*) but does not result in any systematic change in fibre stress–length curve width with increasing actin and myosin filament length.

### 3.3. Work density

**Figure 6.**
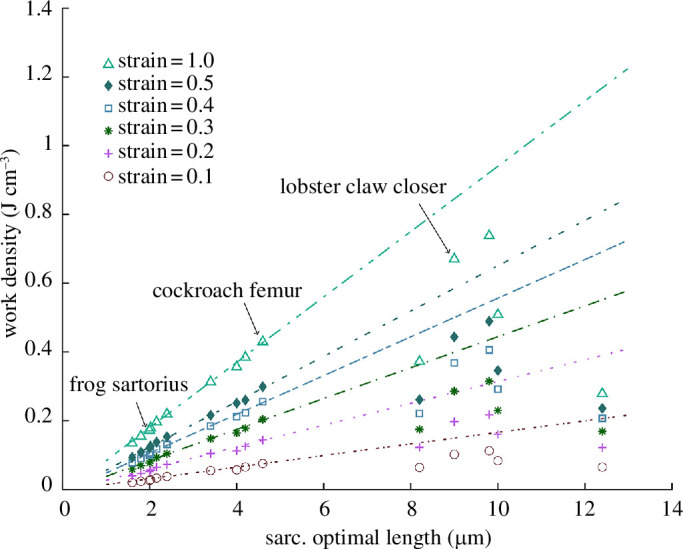
The effect of sarcomere length on muscle fibre work density at different fibre strains for real sarcomeres (symbols) [45,62,65–79] and for idealized sarcomeres (lines). Frog sartorius, cockroach femur and lobster claw closer muscles are highlighted.

The work density of muscle fibres increases with actin and myosin filament lengths for both real and idealized sarcomeres (figure 6); longer actin and myosin filament lengths increase peak stress but leave the fibre stress–length curve width unaffected. Work densities for frog sartorius, cockroach femur and lobster claw closer are 0.17, 0.43 and 0.67 J cm^−3^. Deviation in the $l_{m}/l_{a}$ ratio from $l_{m}={2l}_{a}$ in longer real sarcomeres results in a smaller increase in work density for real than idealized sarcomeres. For example, a short idealized sarcomere with the same $l_{a}$ as the frog sartorius has a predicted work density of 0.18 J cm^−3^, compared to the 0.17 J cm^−3^ predicted for the real sarcomere. However, an idealized sarcomere with the same $l_{a}$ as the lobster claw closer muscle has a work density of 0.84 J cm^−3^, compared to 0.67 J cm^−3^ predicted for the real sarcomere.

## 4. Discussion

Skeletal muscle is a hierarchical structure. Its mechanical performance is an emergent property arising from the molecular agents of contraction, the ultrastructural arrangement of these agents into sarcomeres, and the gross architectural arrangement of sarcomeres into muscle fibres and muscles. We explored the effect of one major aspect of variation in ultrastructure observed only in invertebrates—the length of actin and myosin filaments—on muscle force, displacement and work capacity. We used a minimalist, one-dimensional sliding-filament muscle model [36,40,43] to predict sarcomeric stress–length relationships from actin and myosin filament lengths, and then scaled these results to the muscle fibre level. Increasing actin and myosin filament length increases muscle stress but does not affect muscle strain capacity. Hence, muscles with longer optimum sarcomere length are generally predicted to generate higher peak stresses across comparable displacements, and thus have greater work densities. However, predicted stress and work density depend not only on optimum sarcomere length but also the $l_{m}/l_{a}$ ratio, and deviations from the idealized $l_{m}={2l}_{a}$ at longer optimum sarcomere lengths [45,62,65–79] appear to limit increases in stress and work (figures 4–6). We examine these effects of variation in muscle structure on its mechanical performance, address the limits of the minimalistic model used here, and explore the implications of these predictions for our understanding of sarcomere design.

### 4.1. Muscle structure and mechanical performance

Muscle stress is predicted to increase with increasing actin and myosin filament length for both real and idealized sarcomeres (figure 4). The predicted increase in stress is a direct consequence of the sliding filament theory [28,29,36] in which peak stress is determined by the degree of actin–myosin overlap and the stress per unit overlap (*σ_pl_*) [36]. This predicted increase in stress with increasing actin and myosin filament lengths is empirically supported. Muscle stress increases with increasing sarcomere length across crab species [51,54]. Frog muscles with ~2.5 µm sarcomeres produce stresses of ~34.3 N cm^−2^, barnacle muscles with 7 µm sarcomeres produce 48.0 N cm^−2^, and crayfish muscles with 10 µm sarcomeres produce 64.7 N cm^−2^ [53]. And, there is a strong positive relationship between ‘resting’ sarcomere length and muscle stress [46].

The width of the sarcomeric stress–length relationship is predicted to increase with actin and myosin filament length such that longer sarcomeres can exert large forces over a larger range of sarcomeric displacements (figure 4). However, because an increase in actin and myosin filament, and optimum sarcomere, lengths reduces the number of sarcomeres in series per unit length of muscle fibre, the width of the fibre stress–length curve remains practically unaffected (figure 5). The strain capacity of muscle fibres thus appears to be approximately independent of actin and myosin filament lengths. This is the case for both real and idealized sarcomeres and, unlike stress, this effect does not seem to vary greatly between real and idealized sarcomeres. This invariance of strain capacity, combined with the increase in stress (figures 4 and 5), predicts an increase in the work density ([Fig F5] figure 6). Muscle fibres with longer actin and myosin filaments can, in general, generate larger stresses across an unchanged range of muscle fibre lengths, and therefore presumably joint angles. Unfortunately, there is limited empirical evidence on the variation of force–length curve width and work density across muscles with different actin and myosin filament lengths. The width of fibre or muscle stress–length relationships varies considerably [3,82]. However, our findings suggest that this variation is likely related to factors other than variation in actin and myosin filament length. This is supported by the variation in the width of the force–length curve across vertebrates [3] where actin and myosin filament lengths vary little [43,45,47].

This prediction of a constant muscle fibre strain capacity with increasing actin and myosin filament lengths may be surprising given the observed reduction in muscle fibre velocity with increasing actin and myosin filament lengths [55] attributed to the reduced number of cross-bridges in series [38]. This may be understood by considering displacement from a cross-bridge rather than sarcomere perspective. The maximal active shortening a muscle fibre can undergo ( $x_{f}$ ) is the product of the maximum strain ( $\varepsilon_{f}$ ) and its length ( $L_{f0}$ ). In vertebrate muscle, this small set of parameters is sufficient to describe displacement capacity as the lack of variation in actin and myosin filament lengths means that the relationship between cross-bridges, sarcomeres and fibres is constant. However, the variation in ultrastructure in invertebrates may mean that it is more sensible to instead describe this relationship at the scale of cross-bridges. The displacement capacity of a fibre ( $x_{f}$ ) is then given by the product of the distance between the point of zero actin–myosin overlap and the point at which the myosin contacts the Z-disc (which we define as the product of the displacement capacity of an individual cross-bridge ( $x_{c}$ ) and the number of cross-bridge ‘steps’ that can be taken between these points ( $n_{xc}$ )), the number of sarcomeres per unit length of muscle fibre ( $n_{s}/L_{f0}$ ), and the length of the fibre ( $L_{f0}$ ) (equation (4.1)).


(4.1) $$x_{f}=\left(\frac{x_{c}\;n_{cx}\;n_{s}}{L_{f0}}\right)L_{f0}.$$

For a given muscle length, only one of these parameters is free, i.e. decreasing actin and myosin filament lengths increases the number of sarcomeres in series and vice versa. As a direct consequence, the net effect of ultrastructural changes on strain capacity is zero and, as with vertebrates, displacement capacity can only be altered by changing muscle fibre length. Maximum fibre shortening velocity ( $V_{f}$ ), however, is fibre displacement divided by the product of the time taken for each cross-bridge step ( $t_{c}$ ) and the number of cross-bridge steps that occurs between the point of zero actin–myosin overlap and the point at which the myosin contacts the Z-disc (equation (4.2)). Thus, the maximum shortening velocity is defined solely by the displacement of a single cross-bridge, the number of sarcomeres in series ( $n_{s}$ ), and the time taken for each cross-bridge step (equation (4.3))


(4.2) $$V_{f}=\left(\frac{\left(\frac{x_{c}\;n_{cx}\;n_{s}}{L_{f0}}\right)}{t_{c}\;n_{cx}}\right)L_{f0},$$

(4.3) $$V_{f}=\frac{x_{c}\;n_{s}}{t_{c}}.$$

Cross-bridge step time is defined only by myosin isoform and is as such independent of actin and myosin filament length. Hence, changing actin and myosin filament length can change maximum fibre velocity without changing displacement capacity.

### 4.2. Model limitations

The minimalist sliding-filament model used here considers only two of the three contractile proteins; it omits the role of the large viscoelastic protein titin. The model is also one-dimensional; it considers only actin–myosin overlap along the length of the sarcomere and neglects any variation in sarcomere diameter, lattice structure or dynamic changes in lattice spacing with sarcomere shortening. The model is parametrized with values from empirical studies of vertebrate muscle [36,43,62] with the implicit assumption that these parameters are consistent across a range of actin and myosin filament lengths. These limitations arise directly from the need to simplify muscle to allow for exploration of the effect of the extensive variation in actin and myosin filament lengths on muscle performance. However, we also lack information about the role of these features of muscle in contraction, a knowledge of the variation in these features across muscles, empirically determined force–length relationships for known actin and myosin filament lengths, and an understanding of the mechanisms responsible for the ascending limb of the force–length relationship.

We systematically exclude titin, and the various isoforms of the array of titin-like proteins observed in invertebrates [83–90], from this model. In vertebrates, this large viscoelastic protein [15,25] spans entire half sarcomeres from the Z-disc to M-line [20] and increases stiffness upon muscle activation both by binding to actin [26], and potentially by being wound onto actin filaments by cross-bridge cycling [25,26]. As a result, titin has been suggested to affect muscle force, work and power particularly when the muscle has previously been actively stretched or shortened [15–19,25,91]. The location and size of titin-like proteins appear to vary considerably across invertebrate muscles [83–90], and unlike in vertebrates, very little is known about their active function with the exception of the highly specialized molluscan twitchin [92]. The role of titin in active muscle, and the variation in titin-like proteins likely has implications for our predictions of force and work. However, titin functions as a tunable viscoelastic spring [15], not an active force and work producer [12,23]. Hence, while its omission would likely have critical effects on the predictions of instantaneous force and work during dynamic contractions, it likely would have much less effect on the predicted isometric force and quasi-static work capacity that are the focus of the present study. Even in such contractions, the omission of titin could potentially confound our results if the relative size and function of titin-like proteins varied systematically with other contractile protein lengths. A single study of crayfish claw muscles shows that these long-sarcomere muscles appear to have large titin molecules that could span the longer sarcomeres [87] suggesting that at least in this case, titin could potentially have a similar role across sarcomeres with different optimum lengths. However, this remains a largely unexplored area and a comparison of the predictions of this model to future empirical studies of the shape of the force–length relationship in muscle with different titin-like proteins will provide useful insight into their role in muscle performance.

We use a one-dimensional model that considers only variation in actin and myosin filaments along the length of the sarcomere and fibre. We use a constant *σ_pl_* to describe the slope of the descending limb, implicit in which is the assumption that sarcomere diameter remains constant with increasing actin and myosin filament lengths. An increase in actin and myosin filament lengths at constant sarcomere diameter implies an increase in myosin heads in parallel per unit area. Otten [40] suggested that sarcomere diameter increases linearly with actin and myosin filament length. In this model, this would equate to a systematic increase in *σ_pl_* with increasing actin and myosin filament lengths and result in a prediction of no effect of actin and myosin filament length on stress. However, empirical evidence shows a clear increase in muscle stress with increasing actin and myosin filament lengths [46,51–54]. Although these empirical data should be interpreted with caution, due to difficulties in measuring force at optimum length and in accurately correcting for moment arms and physiological cross-sectional area, the magnitude and consistency of this effect across studies and taxa suggest that muscle stress does increase with actin and myosin filament length. Hence, it appears that the effect of increases in actin and myosin filament length on cross-bridges in parallel, and so force, dominates over any increase in sarcomere diameter. However, based on our understanding of contractile protein and sarcomere structure, we would expect some increase in sarcomere diameter that would result in our model overpredicting increases in stress.

**Figure 7.**
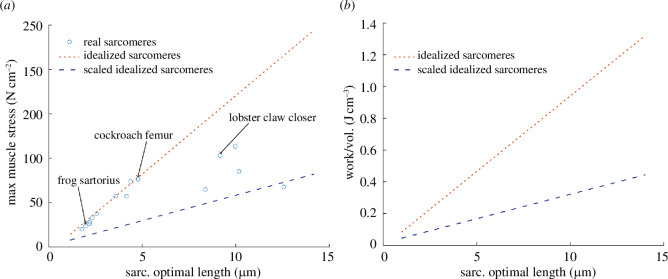
(***a***) Predicted maximum isometric stress for real (blue circles) [45,62,65–79] and idealized (red line) sarcomeres as a function of optimum length, and the same data for idealized sarcomeres after applying max stress correction from empirical data (blue line) [46]. Frog sartorius, cockroach femur and lobster claw closer are highlighted. (***b***) Predicted work density at maximum fibre strain for idealized sarcomeres with unscaled max stress predicted from the model (red line) and scaled max stress adjusted for the slower rate of increase seen in the empirical data (blue line) [46].

An increase in myosin filament length requires the addition of myosin dimers, which may increase filament diameter as more tails are added to the filament core. The staggered arrangement of myosin dimers [14,93] likely minimizes this effect, and myosin filament diameter is practically identical in lobster tail and claw muscles despite a twofold difference in actin and myosin filament length [66]. However, the formation of very long myosin filaments requires the addition of the structural protein paramyosin, which significantly increases filament, and therefore likely sarcomere, diameter [94]. In addition, contractile protein lattice structure varies with optimum sarcomere length, with more actin filaments surrounding each myosin filament as filament length increases [2,45], potentially also increasing sarcomere diameter. To assess the degree to which our assumption of constant sarcomere diameter with increasing actin and myosin filament length may have resulted in an overestimate of stress and work density, we compared the ordinary least squares regression of stress versus sarcomere length from Taylor [46] to our model predictions (figure 7*a*). The stress increment is shallower in the empirical data, suggesting either a concomitant increase in sarcomere diameter, or some other change, with increasing actin and myosin filament length. From this comparison, we derive a correction factor, which confirms that although the predicted increase in work density may be attenuated by factors not accounted for in our model, it is not eliminated (figure 7*b*). Even with the correction, work density increases by almost a factor of four from 0.089 J cm^−3^ in the frog sartorius muscle to 0.35 J cm^−3^ in the lobster claw closer muscle. Interestingly, Taylor’s regression [46] fits the predictions for real sarcomeres [45,62,65–79] much better than those for idealized sarcomeres (figure 7*a*), suggesting that the lower rate of increase in stress with increasing actin and myosin filament length could, at least in part, simply be a result of the apparent change in $l_{m}/l_{a}$ ratio with increasing actin and myosin filament length in real sarcomeres rather than a systematic change in *σ_pl_* that is omitted from our model.

The one-dimensional model used here not only omits sarcomere diameter but also the dynamic changes in lattice spacing that accompany the stretch and shortening of a sarcomere [30]. Here, we assume the dependency of force on sarcomere length to be a consequence of the changing overlap between actin and myosin on the descending limb, and interference from opposing actin and the crumpling of myosin on the ascending limb [36]. However, there is evidence that the change in lattice spacing that must occur during isovolumetric sarcomere stretch and shortening is a major contributor to the variation in force with sarcomere length [30]. Indeed, this effect, as opposed to interference of the opposing actin filament, has been proposed to mechanistically explain the decline in force on the ascending limb of the force–length relationship [61]. If some of the dependence of force on length arises from changes in lattice spacing rather than changes in overlap, this may not be properly described by our model. The use of empirically determined parameters to describe slopes in our model removes the need for a mechanistic understanding but assumes a consistent effect across actin and myosin filament lengths. If lattice spacing is indeed a major contributor to the dependency of force on sarcomere length [30,31,61], then this may not be a valid assumption. Virtually nothing [3,31] is known about how the change in lattice spacing with sarcomere stretch and shortening varies across muscles. However, it is known that muscle lattice structure changes somewhat systematically with actin and myosin filament lengths [2,3,45], which could lead to different effects of sarcomere stretch and shortening on lattice spacing across actin and myosin filament lengths. As with titin and titin-like proteins, a comparison of the predictions of this model to future empirical studies of the shape of the force–length relationship in muscle with different lattice structures will provide useful insight into its role in muscle performance.

In line with previous work [36], we assumed that the steep region of the ascending limb is due to the crumpling of filaments as they contact the Z-disc. However, more recent evidence suggests that the flexural stiffness of myosin may be too high for crumpling to occur and that the structure of the muscle lattice might be such that it allows myosin to pass through the Z-disc [63,64]. This mechanism has been proposed to explain the extremely wide force–length relationships of specialized supercontracting muscles such as those found in the chameleon tongue [95] and blowfly larvae [96]. However, in more typical muscles, it seems that this mechanism only results in a small amount of additional force production at very short muscle lengths in response to very prolonged, largely non-physiological, stimulus durations [97] and, unless some lattice structures limit the ability of myosin to pass through the Z-disc, it is hard to see how this phenomenon would vary with actin and myosin filament lengths. Hence, while this discovery is hugely important for our mechanistic understanding of muscle contraction, and key to understanding the specialization of supercontracting muscle, it seems unlikely to affect the conclusions of the study presented here.

### 4.3. Implications for sarcomere design

We predict an increase in stress and work with increasing actin and myosin filament lengths in both real and idealized sarcomeres. However, deviations from $l_{m}={2l}_{a}$ in longer sarcomeres such that $l_{m}<2l_{a}$ (figure 3) results in a reduced increased in predicted stress and work with increased optimum sarcomere length in real as compared to idealized sarcomeres, sometimes to the extent that large increases in optimum sarcomere length result in little to no increase in stress (figure 4). The predicted increase in stress is not surprising; it has long been theoretically suggested [28,38] and is well-supported empirically [46,51–54]. However, the effect of actin and myosin filament lengths on muscle work density has not, to our knowledge, previously been examined. We examine the implications of the apparent deviation from $l_{m}={2l}_{a}$ in longer sarcomeres, explore the potential functional benefits of an increased work density, and offer suggestions as to why variations in actin and myosin filament length are seemingly restricted to invertebrate muscle.

The comparison of real and idealized sarcomeres presented here suggests there may be an upper limit to the capacity of this structural variation to increase stress and work density. The very limited data available on contractile protein filament lengths [45,62,65–79] suggest that actin and myosin lengths do not continue increasing in parallel; increases in myosin length do not keep pace with increases in actin lengths resulting in sarcomeres in which $l_{m}<2l_{a}$ (figure 3*b*). This should be interpreted with some caution due to the potential for errors in the measurements of actin and myosin filament length arising from issues such as filament shrinkage [43]. However, this deviation from $l_{m}={2l}_{a}$ appears to be seen across a range of studies in long, but not short, sarcomeres [45,62,65–79] (figure 3). It may be that this variation in $l_{m}/l_{a}$ ratio serves some as of yet unknown purpose. For example, it is known that asynchronous flight muscle, not considered here, has long actin filaments relative to myosin despite its relatively short sarcomeres. And, this effect has been related to its high stiffness and low *in vivo* strains [40]. However, it may also be that this lack of continued parallel increase in actin and myosin filament lengths represents a limit to the possibility, or benefits, of continuing to increase filament length. Actin and myosin filaments both have some degree of compliance, and their deformation under cross-bridge force is thought to affect cross-bridge binding [98,99]. If the modulus of the contractile protein filaments remains the same with increasing length, the increased deformation that will occur with increasing length may be too great to retain the spatial relationship between them required for cross-bridge binding. This is supported by the finding that sarcomeres with longer contractile protein lengths have increased actin–myosin packing ratios in their lattice structures [2,45], which presumably spreads cross-bridge load across more actin filaments, and structural reinforcement of myosin with paramyosin [94]. Hence, there may be a limit to the extent to which actin and myosin filament lengths can be increased without disrupting cross-bridge formation. There may also be costs that limit the benefit of greater increases in length. For example, increasing actin–myosin ratios and the addition of paramyosin likely increase sarcomere diameter and so may limit increases in stress with increasing actin and myosin filament lengths beyond some point. However, despite this apparent limit, significant increases in stress and work density are predicted across the range of actin and myosin filament lengths observed (figures 3, [Fig F4] 5 and 6).

It is often held that work density is a mechanical property invariant to anatomical variations [57–60] be it in form of the allocation of a unit of muscle volume into fibre length versus fibre cross-sectional area [5], fibre pennation [100] or joint gearing [101]. This assertion is grounded in the observation that these anatomical ‘design choices’ trade-off force against displacement capacity, and thus have no net effect on work density. The results of this study suggest that there does, however, exist a structural variation that can overcome this seeming universal constraint: increasing actin and myosin filament lengths can leave displacement capacity unaffected, but increase muscle force capacity, and thus muscle work density. Two questions then demand answers. (i) What are the material benefits of an increased work density? (ii) Why are variations in actin and myosin filament length seemingly restricted to invertebrate muscle?

Although an invariant muscle work density is often credited as the origin of limits to muscle mechanical performance [57–60], recent theoretical work has revealed that the energy output of dynamic contractions is often limited by a different characteristic energy instead: because muscle has a maximum absolute shortening speed, v_max_, it cannot supply kinetic energy in excess of its ‘kinetic energy capacity’, *K ~ m v_max_^2^ G^−2^*, where *m* is the mass of the object moved by muscle, and *G* is the gear ratio [102]. Because muscle kinetic energy capacity is typically much smaller than muscle work capacity [102,103], muscle that is functionally specialized to actuate movements that involve large speeds is unlikely to benefit from an increased work density. Instead, a large work density is likely most useful in quasi-static contractions against large external loads, i.e. where muscle workflows into forms of energy distinct from kinetic energy, so that muscle shortening speed capacity and consequently force–velocity trade-offs are of only limited relevance. Two examples may serve as illustrations. First, some small invertebrates such as mantis shrimp, fleas and locusts actuate limb movements not through direct muscle-action, but by storing muscle work temporarily as strain energy through contraction against elastic ‘springs’ [104–107]; this energy is then rapidly released via removal of a ‘latch’ to power explosive movements with peak speeds that exceed what would be achievable by direct muscle contraction by orders of magnitude [107]. In such ‘latch-mediated spring actuation’, the work density of muscle is the limiting parameter [58,59,104]. Consistent with this argument, species of mantis shrimp with longer sarcomeres strike prey faster [108]. Second, feeding is a mechanically demanding task during which muscle does work to fracture food items. Although feeding often involves cyclical muscle contractions, increasing muscle work density may be advantageous for at least three reasons. First, maximizing the work output per contraction reduces the number of necessary cycles and thus minimizes muscle ‘activation’ costs [109], which may often constitute a significant fraction of the total metabolic costs of a contraction [110]. Second, where the force output of a single contraction is increased at no expense to displacement, the work and power output of a single contraction increase simultaneously, so reducing feeding time, and thus dangerous exposure to predators [111,112], and the sometimes extensive metabolic costs of feeding [113]. Third, the range of muscle displacements and therefore joint angles over which muscle can exert large forces is thought to determine the range of food items that can be mechanically processed [114–116]; a simultaneous increase of muscle force and work capacity consequently expands this range.

It is tempting to conclude from these examples that variation in actin and myosin filament length is most prevalent in invertebrates because they are small, so that mechanical feats like rapid movement and feeding are more likely to demand the quasi-static contractions that benefit from an increased work density. Perhaps the functional relevance of these quasi-static contractions is reduced in large animals, because direct muscle actuation becomes more effective [59], because the larger absolute force capacity renders feeding on the same food items less mechanically challenging [117], and because force–velocity trade-offs consequently constrain actin and myosin filament length. Perhaps changes in actin and myosin filament lengths do not primarily serve to increase work capacity to begin with, but target force capacity instead—a particular challenge when increases in muscle volume are constrained by hard exoskeletal shells such as occurs in crab claws [51] or the head capsules of herbivorous insects [6]. And, perhaps our perception of the frequency with which long sarcomeres occur in the ultra diverse invertebrates is biased altogether, because attention has been paid predominantly to extremes in sarcomere ultrastructure, at the expense of a rigorous assessment of what is the ‘norm’. An obvious alternative explanation for the difference in sarcomere design across invertebrates and vertebrates is phylogenetic constraints, though we note that long sarcomeres have been observed across invertebrates as distantly related as ants [118], squid [55] and crabs [46]. Extensive experimental characterization of muscle ultrastructure and mechanical performance across an unbiased sample with maximal phylogenetic diversity will be required to elevate these speculative hypotheses to assertions supported by robust evidence.

## Data Availability

The model is published in its entirety in the paper, all inputs are provided, and we report all data generated.
